# Preparation of Silica Aerogels by Ambient Pressure Drying without Causing Equipment Corrosion

**DOI:** 10.3390/molecules23081935

**Published:** 2018-08-02

**Authors:** Lixiao Zhu, Yali Wang, Suping Cui, Feihua Yang, Zuoren Nie, Qunyan Li, Qi Wei

**Affiliations:** 1 College of Materials Science and Engineering, Beijing University of Technology, 100 Pingleyuan, Chaoyang District, Beijing 100124, China; zhulixiao@emails.bjut.edu.cn (L.Z.); cuisuping@bjut.edu.cn (S.C.); zrnie@bjut.edu.cn (Z.N.); qyli@bjut.edu.cn (Q.L.); 2 State Key Laboratory of Solid Waste Reuse for Building Materials, Beijing 100024, China

**Keywords:** silica aerogel, corrosion, silylation agent, ambient pressure drying

## Abstract

The silica aerogels were prepared via a sol-gel technique and ambient pressure drying by using industrial solid wastes, dislodged sludges, as raw materials. A strategy was put forward to reduce the corrosion of equipment during the drying procedure. The pore structure, hydrophobicity, and thermal insulation property of the obtained samples were investigated in detail. The results show that the corrosion can be effectively avoided by using an equimolar mixture of trimethylchlorosilane (TMCS) and hexamethyldisilazane (HMDS) as silylation agents. At a Si:TMCS:HMDS molar ratio of 1:0.375:0.375, the silica aerogels possess a desirable pore structure with a pore volume of 3.3 ± 0.1 cm^3^/g and a most probable pore size of 18.5 nm, a high hydrophobicity with a water contact angle of 144.2 ± 1.1°, and a low thermal conductivity of 0.031 ± 0.001 W/(m∙K).

## 1. Introduction

Silica aerogel is a kind of promising materials for thermal insulation due to its extremely low thermal conductivity (~0.04 W/(m∙K)) originated from low density (<0.1 g/cm^3^), high porosity (~99%), and adjustable nanoporous structure, together with its strong resistance to burning [1,2,3,4,5]. Generally, silica aerogel is produced by a sol-gel technique with silicon alkoxides, such as tetraethoxysilane (TEOS) [6,7] or tetramethoxysilane (TMOS) [8,9] as precursors. Recently, sodium silicate [10,11,12] or solid wastes such as oil shale ash [13], fly ash [14], bagasse ash [15], and rice husk ash [16] have also been used as raw materials to prepare silica aerogels. Drying process, including either supercritical drying (SD) [17] or ambient pressure drying (APD) [18,19], plays a critical role in the manufacture of silica aerogels. Ambient pressure drying has attracted a great deal of attention in recent years to reduce the production risk and equipment cost. Before ambient pressure drying, liquids with high surface tension in the pores within the wet gels should be replaced by solvents with low surface tension to reduce the capillary pressure, which significantly damages the pore structure of silica aerogel during the drying procedure. Additionally, surface silylation of silica gels with alkyl groups is required to eliminate the terminal silanols (hydroxyl groups, -SiOH) as much as possible, which cause the gels to shrink via irreversible condensation during ambient pressure drying [15]. Table 1 summarizes the range of thermal and textual properties of silica aerogels that are produced from different raw materials and drying processes.

Trimethylchlorosilane (TMCS) [20,21] is the most common organosilicone monomer that is usually used as silylation agent in the preparation of silica aerogels. During the drying procedure, the reaction between surface terminal hydroxyl groups and TMCS generates acidic vapor (HCl), leading to a severe damage to the stainless steel chamber of drying equipment since the corrosive vapor can not be completely removed before drying. Therefore, it is of great importance to reduce the release of corrosive vapor in order to avoid equipment corrosion and serious pollution to the environment during the drying procedure. It is expected that if an equimolar mixture of TMCS and hexamethyldisilazane (HMDS) [22,23] is used for silylation, the corrosive vapor would be less yielded because HCl will be neutralized by NH_3_, resulting from the reaction between surface terminal hydroxyl groups and HMDZ. In the previous work, Rao et al. has prepared low-density silica aerogels through the use of the combination of silylation agents, for example, HMDS + TMCS, HMDSO (hexamethyldisiloxane) + TMCS or HMDSO + HMDS [24]. However, the equipment corrosion behavior caused by the corrosive vapor during drying process has not yet been investigated. Additionally, sodium silicate rather than solid waste is used for silica source in their work and the thermal insulation property has not been detected.

In the present work, silica aerogels were prepared by ambient pressure drying with solid wastes—dislodged sludges as raw materials. The corrosion caused by releasing HCl vapor on stainless steel slice as a representative of drying equipment chamber is investigated in detail. An equimolar mixture of HMDS and TMCS was used as silylation agent to avoid equipment corrosion by reducing the release of corrosive vapors. The amount of silylation agent was optimized to obtain silica aerogels with desirable pore structure and thermal insulation property. To the best of our knowledge, similar work has not yet been reported previously.

## 2. Results and Discussion

**Figure 1:**
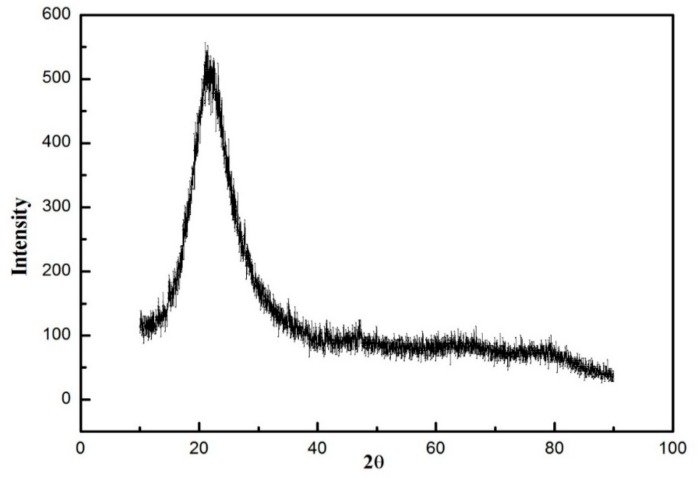
X-ray diffraction (XRD) pattern of dislodged sludge.

A total of five samples were prepared and denoted as 1–5, according to the Si/TMCS/HMDS molar ratio of 1:1:0, 1:0.5:0.5, 1:0.375:0.375, 1:0.25:0.25, and 1:0.125:0.125, respectively. The corrosion behavior was revealed by using samples 1 and 2. The samples 2, 3, 4, and 5 were designed to optimize the amount of mixed silylation agents. The raw material, dislodged sludge, is a kind of industrial by-product and its composition detected by X-ray fluorescence (XRF) is listed in Table 2. It can be seen that dislodged sludge is predominantly composed of silica (up to 99.37 ± 0.02 wt %), together with minor impurities, such as MgO, Fe_2_O_3_, MgO, and so on. The dislodged sludge is amorphous, as shown by the XRD pattern in Figure 1, where only a broad peak, characteristic for non-crystalline solid, is observed.

### 2.1. Corrosion Behaviour under Different Drying Atmosphere

**Figure 2:**
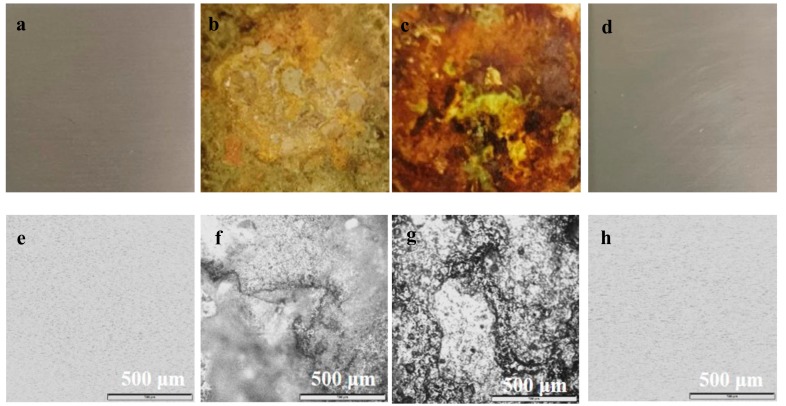
Digital photos (**a**–**d**) and optical microscope images(**e**–**h**) of the stainless steel slice exposed to different drying atmosphere for different time (**a**,**e**: fresh steel; **b**,**f**: trimethylchlorosilane (TMCS) as sole silylation agent, 36 h; **c**,**g**: TMCS as sole silylation agent, 60 h; **d**,**h**: equimolar mixture of TMCS and hexamethyldisilazane(HMDS) as silylation agent, 36 h).

Figure 2 show the digital photos and optical microscope images of the stainless steel slices that were exposed to different drying atmosphere for different time. Serious rust can be observed on the surface of the steel exposed to the drying atmosphere with TMCS as sole silylation agent. The steel turns into grey and yellow with rough dirty surface covered by irregular granules and cracks (Figure 2b,c). A gradual destruction of the stainless steel can also be observed from the OM images (Figure 2f,g). This observation indicates that the drying equipment can be severely corroded and damaged by the vapor releasing during the drying procedure for the use of sole TMCS silylation agent, and the corrosion is greatly enhanced with an increasing exposure time. On the contrary, the steel that was exposed to the atmosphere for the synthesis with an equimolar mixture of TMCS and HMDS as silylation agent remains almost intact (Figure 2d,h), with a smooth and clean surface as that of fresh steel (Figure 2a,e), indicating that corrosion does not take place.

**Figure 3:**
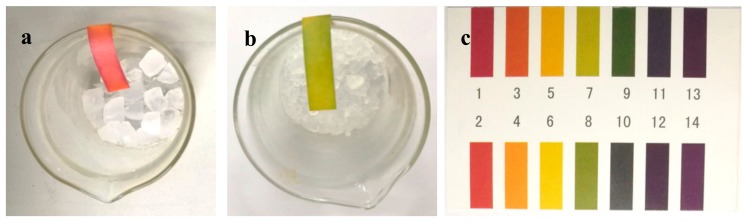
Nature of the vapors generated during the drying procedure of the samples. (**a**) sample 1; (**b**) sample 2; and (**c**) the standard colorimetric card.

In order to further investigate the reason for corrosion, the nature of the vapors that are generated from sample 1 and 2 with a molar ratio of Si:TMCS:HMDS of 1:1:0, and 1:0.5:0.5, respectively, is detected by pH-indicator papers during the drying procedure. As shown in Figure 3a, the vapor is acidic when sole TMCS is used. However, neutral vapor is released for the use of equimolar mixture of TMCS and HMDS, as evidenced by the color approximately matching to a pH value of 7 (Figure 3b).

The generation of vapor can be indicated by the following chemical equations. For the silylation with sole TMCS, when TMCS is mixed with ethanol, alcoholysis takes place to yield HCl and intermediate species (CH_3_)_3_SiOC_2_H_5_ (Equation (1)) [25], and then CH_3_ groups will be terminally linked to the surface of aerogels via the condensation reaction between –OC_2_H_5_ and the surface –OH of silica aerogels (Equation (2)) [26,27]. The release of strongly corrosive HCl leads to a serious corrosion on the stainless steel, as shown in Figure 2b,c.

(1) $${\left({\mathrm{CH}}_{3}\right)}_{3}\mathrm{SiCl}+{\mathrm{CH}}_{3}{\mathrm{CH}}_{2}\mathrm{OH}\to {\left({\mathrm{CH}}_{3}\right)}_{3}{\mathrm{SiOCH}}_{2}{\mathrm{CH}}_{3}+\mathrm{HCl},$$

(2) $$-\mathrm{SiOH}+{\left({\mathrm{CH}}_{3}\right)}_{3}{\mathrm{SiOCH}}_{2}{\mathrm{CH}}_{3}\to -\mathrm{Si}-O-\mathrm{Si}{\left({\mathrm{CH}}_{3}\right)}_{3}+{\mathrm{CH}}_{3}{\mathrm{CH}}_{2}\mathrm{OH},$$

For the silylation with an equimolar mixture of TMCS and HMDS, the procedure is much more complicated than that with a sole silylation agent (TMCS). An alcoholysis also occurs when HMDS is mixed with ethanol, generating NH_3_ and (CH_3_)_3_SiOC_2_H_5_ (Equation (3)). White NH_4_Cl precipitates appear immediately as soon as the mixture of TMCS and HMDS is added into the mixture of ethanol and hexane, according to the reaction between NH_3_ and HCl (Equation (4)). After removing NH_4_Cl precipitates by filtration, the filtrate is used for silylation following the same procedure as that with a sole silylation agent (Equation (2)). Since strongly corrosive HCl has been neutralized by NH_3_, no corrosion is observed on the stainless steel, as shown in Figure 2d. Although NH_4_Cl is a kind of weakly acidic salt, its aquous solution is much less acidic than that of HCl. Additionally, unlike HCl, NH_4_Cl is non-volatile. More importantly, a majority of NH_4_Cl has been removed before silylation.

(3) $${\left({\mathrm{CH}}_{3}\right)}_{3}\mathrm{SiN}\mathrm{HSi}{\left({\mathrm{CH}}_{3}\right)}_{3}+2{\mathrm{CH}}_{3}{\mathrm{CH}}_{2}\mathrm{OH}\to 2{\left({\mathrm{CH}}_{3}\right)}_{3}{\mathrm{SiOCH}}_{2}{\mathrm{CH}}_{3}+{\mathrm{NH}}_{3},$$

(4) $$\mathrm{HCl}+{\mathrm{NH}}_{3}\to {\mathrm{NH}}_{4}\mathrm{Cl},$$

It has been proved that an equimolar mixture of TMCS and HMDS as silylation agents is able to effectively reduce equipment corrosion, however, further work is also necessarily required to reveal the effect of the amount of silylation agents on the pore structure, hydrophobicity, and thermal conductivity of the obtained materials. Since silylation reagents (particularly HMDS) are expensive, it is considerably necessary to decrease the amount of silylation reagents from the viewpoint of costs. However, insufficient silylation reagents lead to abundant residual silanols on the surface of wet gels, causing the collapse of pore structure, and consequently, the deterioration of the thermal insulation properties of silica aerogels. Therefore, it is of great importance to optimize the dosage of silylation reagents. In order to obtain the optimal amount, the molar ratio of silylation agents is designed to decrease proportionally, and then the corresponding pore structure, hydrophobicity, and thermal conductivity are investigated in detail in the following sections.

### 2.2. Pore Structure

**Figure 4:**
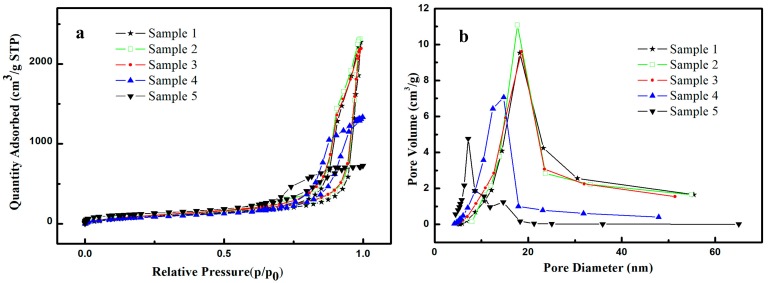
N_2_ adsorption–desorption isotherms (**a**) and pore size distributions (**b**) of silica aerogels with different f silylation agents.

Figure 4 shows the N_2_ adsorption-desorption isotherms and pore size distributions of the samples using different silylation agents. A type IV isotherm with a clear hysteresis loop is observed in all samples, indicating that the samples have a mesoporous structure. It can be seen from Figure 4b that the most probable pore size of the samples is about 7.1–18.2 nm. The samples also display a large shallow shoulder, ranging from 10 to 70 nm, which may be attributed to the larger pores that are derived from the aggregation and packing of larger particles. The nitrogen adsorption capacity of the sample 1 and 2 is up to 2500 cm^3^/g (STP), confirming that the sample 1 and 2 have a high porosity. The isotherms and pore size distributions for sample 1 and sample 2 almost overlap with each other, respectively. The textural parameters of the samples are listed in Table 3. As shown in the table, sample 1 and 2 possess a similar pore volume and pore size, therefore it can be deduced that using HMDS to neutralize HCl has a negligible effect on the pore structure. This observation can be explained on the basis of the following statement. The amount of hydroxyl groups that are consumed by silylation depends on the amount of leaving groups, for instance, Si-Cl or Si-NH, of the silylation agents. In the present work, the molar ratio of TMCS or the mixture of TMCS and HMDS used for silylation is the same in both sample 1 and 2, so that the amount of residual hydroxyl groups on the surface of sample 1 and 2 after silylation is almost equal to each other. This means that, for sample 1 and 2, the condensation of wet gels via hydroxyl groups occurs similarly, thus leading to a similar pore structure [28].

The nitrogen adsorption capacity of silica aerogels decreases with decreasing amount of the mixture of silylation agents, except for the sample with a Si:TMCS:HMDS molar ratio of 1:0.375:0.375 (sample 3). Correspondingly, the pore volume and pore size for sample 3 change very slightly compared to those of sample 2, indicating that the pore structure remains almost intact for sample 3. The adsorption capacity goes down to only half of that of sample 2 and the pore volume and pore size also decrease considerably when the Si:TMCS:HMDS molar ratio drops to 1:0.25:0.25 (sample 4). Further decrease of silylation agents brings about a dramatical reduction of porosity, resulting in a pore volume of only 1.1 ± 0.1 cm^3^/g and a pore size of 7.1 nm, less than one-third of that of sample 2, respectively. Table 3 also exhibits the bulk density of the silica aerogels. The bulk density of samples 1–3 ranges from 0.10 ± 0.01 to 0.11 ± 0.01 g/cm^3^, but that for sample 4, it is much higher due to the shrinkage. A high surface area remains in sample 5, despite that the pore volume is the lowest, which is probably owing to the small pore size. For a porous material with a fixed porosity, pores with smaller size tend to provide larger surface area.

### 2.3. Morphology and Hydrophobicity

**Figure 5:**
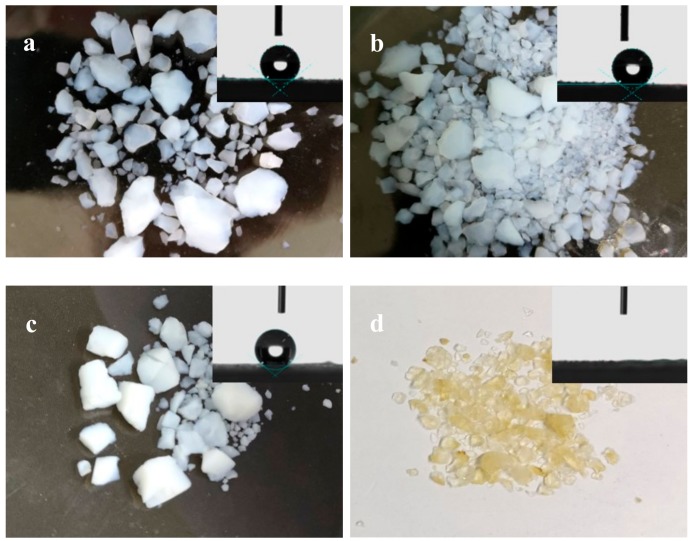
The digital photos and the shapes of water droplets on the surface of the samples (insets). (**a**) sample1, (**b**) sample 2, (**c**) sample 3, and (**d**) sample 5.

Figure 5 shows the morphology and the shape of water droplets on the surface of representative samples and Table 4 lists the water contact angles for all samples. Sample 1–3 consists of granular piece and small monoliths, which are the typical morphology of silica aerogels prepared by ambient pressure drying. However, a severe shrinkage occurs for the sample 5, indicating a failure to obtain silica aerogels [29]. As shown in the insets of Figure 5, water drops stand on the surface with a contact angle of 147.3 ± 1.1°, 145.6 ± 1.4°, and 144.2 ± 1.1° for the sample 1 to 3, respectively, indicating that the samples are hydrophobic, regardless of the silylation agents. However, the water drop spreads rapidly on the surface of sample 5, revealing that the sample is hydrophilic, as demonstrated by a contact angle of 0°.

**Figure 6:**
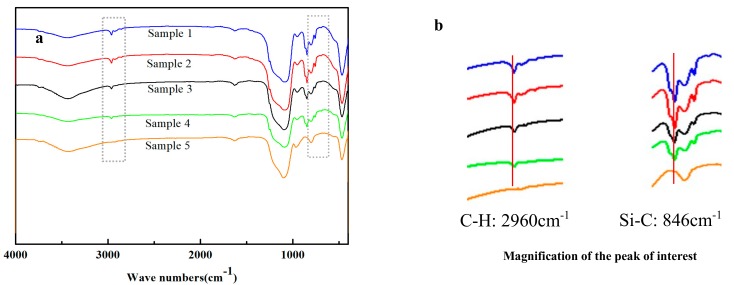
FT-IR spectra of silica aerogels (**a**) and the magnification of the peaks of interest (**b**).

**Figure 7:**
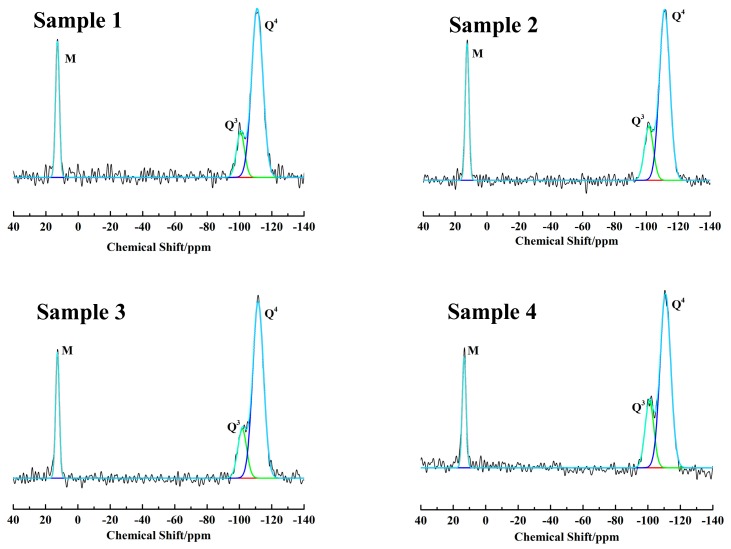

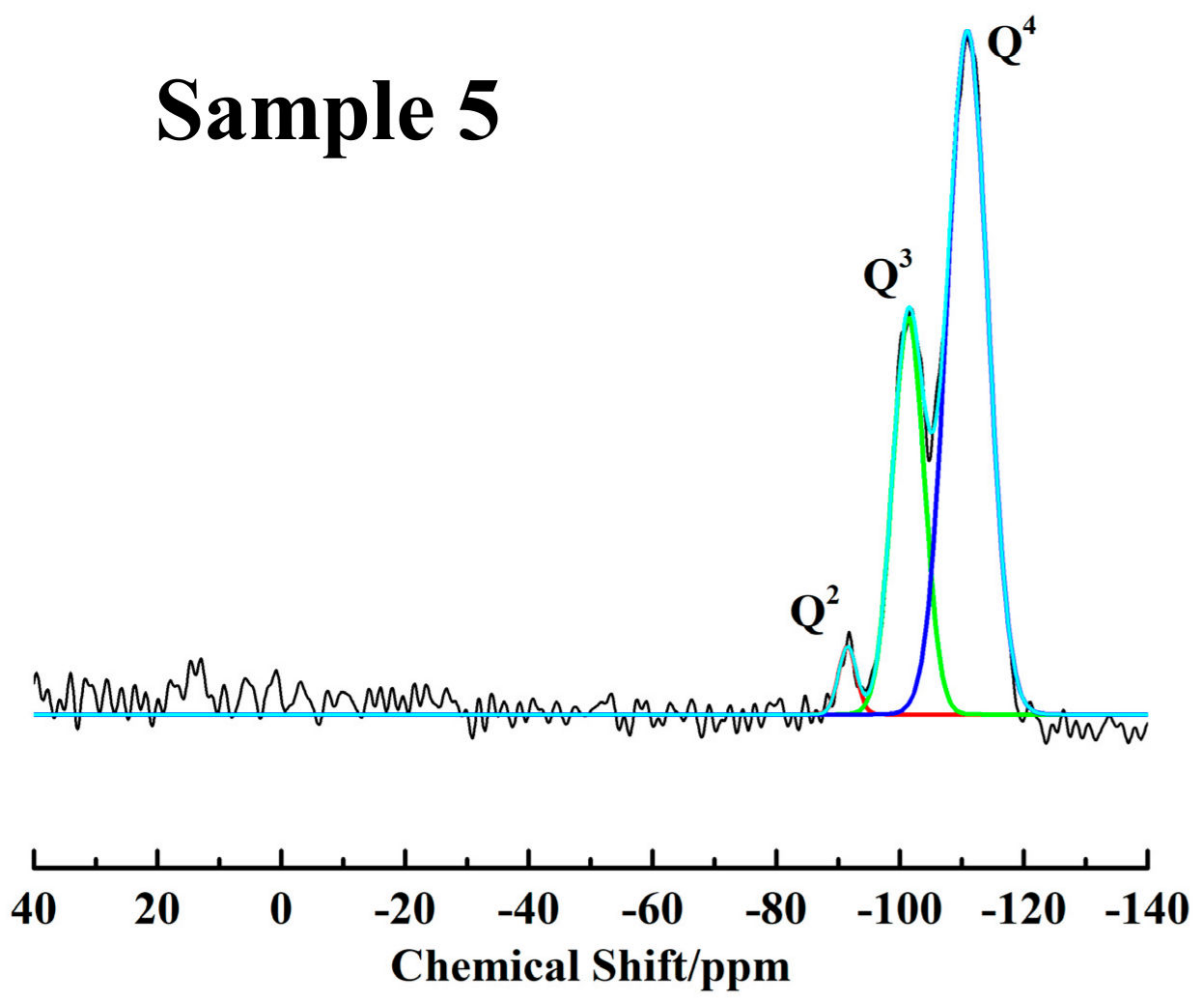
Solid state ^29^Si MAS NMR spectra of silica aerogels fitted by Gaussian approach.

The hydrophobicity of silica aerogels is ascribed to the successful silylation of -CH_3_. During the surface silylation, Si-OH reacts with the silylation agent, and then the hydrophobic -CH_3_ group replaces the hydrophilic Si-OH, thus making the gel hydrophobic. The successful silylation can be confirmed by FT-IR spectra (Figure 6) and solid state ^29^Si MAS NMR spectra (Figure 7). As shown in Figure 6, the absorption peak at 2960 cm^−1^ can be assigned to the vibration C-H, and the peak at 846 cm^−1^ can be attributed to Si-C [30]. However, the above-mentioned absorption bands are absent in sample 5. The presence of CH_3_ groups in silica aerogels can be further confirmed by solid state ^29^Si MAS NMR. As shown in Figure 7, the silicon atoms Q^4^[Si(OSi)_4_], Q^3^[Si(OSi)_3_(OH)], Q^2^[Si(OSi)_2_(OH)_2_], and M[Si(OSi)(CH_3_)_3_] display a resonance at a chemical shift of −112, −100, −90, and 12 ppm [31], respectively. However, the resonance M completely disappears for the sample 5, consistent with the FT-IR spectrum, revealing that silica aerogels have not been successfully silylated at such a low concentration of silylation agents. As a result, the silica aerogels become hydrophilic with a water contact angle of 0°.

The amount of CH_3_ groups and surface silanols in the samples can be quantitatively calculated from the Gaussian-fitted resonance M and Q (Q^3^ + Q^2^), respectively. The relative amount (mol %) of different types of silicon atoms can be calculated by integrating the Q^m^ and M resonances and the results are summarized in Table 4. The absolute values (mmol/g) for the Si-OH and Si-CH_3_ in the samples can be determined based on the following formula [32], where the item I represents the molar concentration (mol %) of Q^m^ (m = 2–4) and M species.

(5) $$\left[\mathrm{OH}\right]=\frac{I_{Q^{3}}+2\times I_{Q^{2}}}{60\times I_{Q^{4}}+69\times I_{Q^{3}}+78\times I_{Q^{2}}+81\times I_{M}}\times 1000,$$

(6) $$\left[{\mathrm{CH}}_{3}\right]=\frac{3\times I_{M}}{60\times I_{Q^{4}}+69\times I_{Q^{3}}+78\times I_{Q^{2}}+81\times I_{M}}\times 1000,$$

It can be seen from Table 4 that the concentration of methyl groups decreases from 8.5 ± 0.1 for sample 2 to 0 mmol/g for sample 5, and that of surface hydroxyl groups increases from 2.6 ± 0.1 for sample 2 to 5.7 ± 0.1 mmol/g for sample 5. The absence of methyl groups and the considerably high concentration of surface hydroxyl groups are responsible for the hydrophilic property for the sample with a Si:TMCS:HMDS molar ratio of 1:0.125:0.125 (sample 5). It is noticed that the water contact angles are inversely proportional to the concentration of residual hydroxyl groups and are proportional to the methyl concentration on the gel surface.

### 2.4. Thermal Conductivity

The thermal conductivity of the samples is listed in Table 3. It can be seen that the thermal conductivity of the sample 2 modified by a mixed silylation agent remains almost constant with that of the sample 1 silylated by sole TMCS, which is probably due to their similar pore structure. Table 3 also compares the thermal conductivity of silica aerogels with a different molar ratio of mixed silylation agents (Sample 2–5). The thermal conductivity of the samples increases with decreasing concentration of the silylation agents. The thermal conductivity of sample 5 (0.087 ± 0.001 W/(m∙K)) is much higher than that of other samples because of the severe collapse of the pore structure. The failure of surface silylation gives rise to more shrinkage during the drying of the gels due to the condensation of the hydroxyl groups (Figure 5d), resulting in a more deteriorated pore structure. The samples 2 and 3 have lower thermal conductivities, predominantly benefiting from the desirable mesoporous structure. It is generally believed that conduction and convection are the main modes for heat transfer [33,34]. Conduction can be considerably reduced in highly porous materials because the pathways for heat transfer through the solid framework are very limited. Additionally, convective gaseous heat transfer is also effectively restricted in silica aerogels with a pore size that is much smaller than the mean free path of air molecules (60–70 nm). The concentration of silylation agents in sample 3 is less than that in sample 2, but the thermal conductivity remains almost the same, so the optimal amount of silylation agents is 1:0.375:0.375 (Si:TMCS:HMDS molar ratio). Table 5 compares the thermal conductivity between our samples and other thermal insulation materials. The thermal conductivity of our samples, no matter what kind of silylation agents is used, is much lower than those of other thermal insulation materials in previous work, revealing a superior thermal insulation property of our samples (Table 3 and Table 5). A similar trend is also observed for the silica aerogels silylated with sole TMCS, as reported in our previous work [28], where reducing the mole ratio of Si:TMCS from 1:1 to 1:0.5 results in a slight increase of thermal conductivity (from 0.030 to 0.033 W/(m∙K)), but a further decease to 1:0.25 leads to a double thermal conductivity (0.060 W/(m∙K)) since the pore volume is nearly twice decreased. Such a high thermal conductivity is unfavorable for silica aerogels and therefore reducing the ratio of Si:TMCS to less than 1:0.25 is not encouraged.

## 3. Materials and Methods

### 3.1. Materials

The dislodged sludge, which is a kind of industrial by-product (or industrial solid waste), was obtained from Yunnan Kiewitt New Materials Co., Ltd., Kunming, China. Hexane (98%), Ethanol (EtOH, 99.8%), Sodium hydroxide (NaOH, 96%), and aqueous ammonia (NH_3_·H_2_O, 26%) were provided by Beijing Chemical Works (Beijing, China). 732 cation exchange resin, trimethylchlorosilane (TMCS, 98%) and hexamethyldisilazane (HMDS, 98%) were purchased from Beijing HWRK Chem Co., LTD. (Beijing, China). A water purification system (Ulupure, Chengdu, China) is used to produce deionized water (H_2_O).

### 3.2. Sample Preparation

The dislodged sludge was pre-treated based on our previous work [28]. The preparation procedure of silica aerogels was described, as follows. 10 g pre-treated dislodged sludge and 90 mL NaOH solution (3 M) were mixed evenly. The mixture was heated to 100 °C and then refluxed for 4 h. After suction filtration, the filtrate was ion-exchanged through 732 cation exchange resin to obtain a silica sol with a pH of around 2. The pH of the sol was adjusted to 5 with aqueous ammonia (1 M) and the silica wet gel (hydrogel) was formed in 5 min. The wet gel was aged in deionized water at 50 °C for 6 h in order to strengthen the network. The water was decanted out and the residual water was exchanged with ethanol at 50 °C for two times, each time for 4 h. Afterwards, the hexane was used to replace the ethanol for 4 h at 50 °C. For the silylation with a sole silylation agent TMCS, the gel was immersed in a mixture of hexane, ethanol, and TMCS for 24 h at 50 °C, respectively, and then followed by a wash with hexane for 24 h at 50 °C to remove the residual silylation agent. For the silylation with an equimolar mixture of TMCS and HMDS, a mixture of TMCS and HMDS was dissolved in a mixture of hexane and ethanol. White precipitate was observed in a few minutes, and after filtration the filtrate was used for silylation, following the same procedure as that with a sole silylation agent. Finally, the solvent was removed and the gel was dried under atmospheric pressure at 50 and 200 °C for 1 h, respectively, with a constant heating rate of 20 °C/h. A total of five samples were prepared and denoted as 1–5, according to the Si/TMCS/HMDS molar ratio of 1:1:0, 1:0.5:0.5, 1:0.375:0.375, 1:0.25:0.25, and 1:0.125:0.125, respectively.

### 3.3. Characterization

The chemical composition of dislodged sludge was analyzed by X-Ray Fluorescence (XRF-1800, Shimadzu, Kyoto, Japan). Powder X-ray diffraction (XRD) pattern was recorded on an XRD-7000 (Shimadzu, Kyoto, Japan) diffractometer using a high power Ni-filtered Cu-Kα radiation (1.541 Å) source. The pore structure of silica aerogels (0.1 g) was determined by the ASAP 2020M volumetric adsorption analyzer (Micromeritics, Atlanta, GA, USA). Before measurement, silica aerogels were degassed at 200 °C for 3 h in order to remove the impurities or moisture. The specific surface area was calculated using the BET equation and the pore size distribution was obtained by the Saito-Foley modified BJH method based on the physical parameters appropriate for mesoporous silica materials. The pore volume was measured at the saturated pressure (*P*/*P*_0_ = 0.99). The bulk density was detected by measuring the mass (by a microbalance with 10^−5^ g accuracy) and volume of silica aerogels [37]. The water contact angle was measured by the OCA20 video-based contact angle system (Dataphysics, Filderstadt, Germany). A picture of a water droplet of 8 μL, injected on the sample surface with a rate of 2 μL/S, was taken by a camera for water contact angle calculation. The morphology of the stainless steel slice exposed to different drying atmosphere was observed by optical microscope (Olympus-PMG3, Olympus Corporation, Shibuya, Japan). The nature of the vapors can be determined by the variation of the color as the vapors pass through the wet pH-indicator papers. Infrared spectra were acquired with a 5700 FT-IR spectroscopy (Nicolet, Madison, WI, USA) in a wavenumber range of 400 to 4000 cm^−1^ while using aerogel powder diluted with potassium bromide (KBr) pellets. The thermal conductivity was measured with the thermal constants analyzer (TPS 3500, Hot Disk, Göteborg, Sweden), with an accuracy of 0.001 W/(m∙K) using a transient plane heat source method by immersing the probe completely into the silica aerogel powder at room temperature. Solid state ^29^Si MAS NMR spectra were detected by the 400 MHz WB Solid-State NMR spectrometer (Bruker, Fällanden, Switzerland), with a spinning rate of 9 kHz, a 90° pulse length of 4.2 µs, and a delay time of 35 s. 1600 scans were used to accumulate the signals. Chemical shifts were referenced to TMS at 0 ppm. All of the measurements in this paper were repeated three times. The data in all the tables are the average of multiple measurements and standard deviation.

## 4. Conclusions

Silica aerogels have been successfully prepared using dislodged sludge as silica source via ambient pressure drying. The equipment corrosion can be effectively avoided by using an equimolar mixture of trimethylchlorosilane (TMCS) and hexamethyldisilazane (HMDS) as silylation agent, where NH_3_ is expected to neutralize corrosive vapor HCl. The optimal molar ratio of Si:TMCS:HMDS is 1:0.375:0.375 and silica aerogels prepared under such a condition have a desirable pore structure with a pore volume of 3.3 ± 0.1 cm^3^/g and a most probable pore size of 18.5 nm, a high hydrophobicity with a water contact angle of 144.2 ± 1.1°, and a low thermal conductivity of 0.031 ± 0.001 W/(m∙K).

## Figures and Tables

**Table 1:** Thermal and textual properties of silica aerogels produced from different raw materials and drying processes.

Precursor	Drying Process	Pore Volume (cm^3^/g)	Pore Size (nm)	Thermal Conductivity (W/(m∙K))	Reference
TEOS	SD	3.93–4.25	25–75	0.008–0.030	[6]
TMOS	SD	-	10–25	0.021–0.065	[8]
Sodium silicate	APD	1.42–2.10	5–30	0.059–0.098	[10]
Oil shale ash	APD	2.37–2.77	13.1–14.1	-	[13]
Fly ash	APD	0.078–4.875	7.69–24.09	-	[14]
Bagasse ash	APD	0.75–2.13	3.40–3.89	-	[15]
Rice husk ash	APD	0.46–0.78	8.3–9.8	-	[16]

**Table 2:** X-ray Fluorescence (XRF) results of dislodged sludge.

Component	SiO_2_	MgO	Fe_2_O_3_	CaO	K_2_O	SO_3_	Na_2_O
Concentration (wt %)	99.37 ± 0.02	0.26 ± 0.03	0.19 ± 0.01	0.08 ± 0.02	0.04 ± 0.02	0.02 ± 0.01	0.02 ± 0.01

**Table 3:** Textural parameters and thermal conductivities of silica aerogels with different silylation agents.

Samples	Si:TMCS:HMDS (Molar Ratio)	P_b_ (g/cm^3^)	S_BET_ (m^2^/g)	V (cm^3^/g)	D_P_ (nm)	λ (W/(m∙K))
1	1:1:0	0.10 ± 0.01	372 ± 2	3.5 ± 0.1	18.2	0.030 ± 0.001
2	1:0.5:0.5	0.10 ± 0.01	406 ± 4	3.5 ± 0.2	17.6	0.030 ± 0.001
3	1:0.375:0.375	0.11 ± 0.01	410 ± 5	3.3 ± 0.1	18.5	0.031 ± 0.001
4	1:0.25:0.25	0.16 ± 0.01	319 ± 2	2.0 ± 0.1	14.7	0.044 ± 0.002
5	1:0.125:0.125	-	457 ± 3	1.1 ± 0.1	7.1	0.087 ± 0.001

P_b_, S_BET_, V, D_P_, and λ denote the bulk density, specific surface area, pore volume, most probable pore size and thermal conductivity, respectively.

**Table 4:** Solid state ^29^Si MAS NMR fitting results and water contact angles of silica aerogels.

Sample	I_M_ (mol %)	I_Q_^4^ (mol %)	I_Q_^3^ (mol %)	I_Q_^2^ (mol %)	[OH] (mmol/g)	[CH_3_] (mmol/g)	WCA (°)
1	18.9 ± 0.1	67.1 ± 0.1	14.0 ± 0.1	0	2.1 ± 0.1	8.7 ± 0.1	147.3 ± 1.1
2	18.6 ± 0.1	64.2 ± 0.1	17.2 ± 0.1	0	2.6 ± 0.1	8.5 ± 0.1	145.6 ± 1.4
3	16.8 ± 0.1	67.6 ± 0.1	15.6 ± 0.1	0	2.4 ± 0.1	7.7 ± 0.1	144.2 ± 1.1
4	15.0 ± 0.1	64.6 ± 0.1	20.4 ± 0.1	0	3.2 ± 0.1	6.9 ± 0.1	136.3 ± 0.9
5	0	66.8 ± 0.1	30.3 ± 0.1	2.9 ± 0.1	5.7 ± 0.1	0	0

**Table 5:** Comparison of the thermal conductivity of different thermal insulation materials.

Materials/Medium.	Thermal Conductivity * (W/(m∙K))	Reference
Static dry air	0.025	[35]
Perlite	0.052–0.056	[36]
Glass fiber	0.042–0.046	[36]
Rock wool	0.040–0.049	[36]
Sample 3	0.030–0.032	This work

* The thermal conductivity was measured with the Hot Disk thermal constants analyzer using a transient plane heat source method.
